# A Case of Psoriasis Vulgaris Treated with Brodalumab in a Hemodialysis Patient with End-Stage Renal Disease due to Diabetic Nephropathy

**DOI:** 10.1155/2020/3863152

**Published:** 2020-02-14

**Authors:** Masafumi Ishibashi, Rie Shiiyama

**Affiliations:** Department of Dermatology, Nippon Kokan Hospital, Kawasaki, Japan

## Abstract

Psoriasis vulgaris is not frequently seen in patients on hemodialysis. However, these patients have limited treatment for psoriasis due to concerns about complications. We report the case of a psoriatic patient with end-stage renal disease on hemodialysis, safely treated with brodalumab. A 60-year-old man who presented with a 20-year history of recalcitrant severe psoriasis. He had diabetes from 40 years ago, and hemodialysis was initiated due to the progression of renal dysfunction two months ago. He was treated with brodalumab, and skin lesions improved markedly. He began to have a chronic cough four months after starting brodalumab. CT showed diffuse ground-glass shadow and pleural effusion in both lungs. Transbronchial lung biopsy showed no findings suggestive of interstitial pneumonia. In bronchoalveolar lavage fluid, mycobacteria and fungi were not identified. The T-SPOT.TB test was negative. It was considered to be a symptom of overflow due to excessive fluid volume based on insufficient dietary management. Brodalumab was continued, and respiratory symptoms improved with proper weight setting and adequate dietary control. No recurrence of rash has been seen 12 months after the initiation of brodalumab. There were no serious adverse events.

## 1. Introduction

There have been several case reports of safe and effective biological treatment for psoriasis patients undergoing hemodialysis, with many cases treated with ustekinumab or adalimumab [1–3]. Recently, there was a report of a psoriasis patient on hemodialysis treated with anti-IL-17A antibody [4, 5].

## 2. Case Presentation

**Figure 1:**
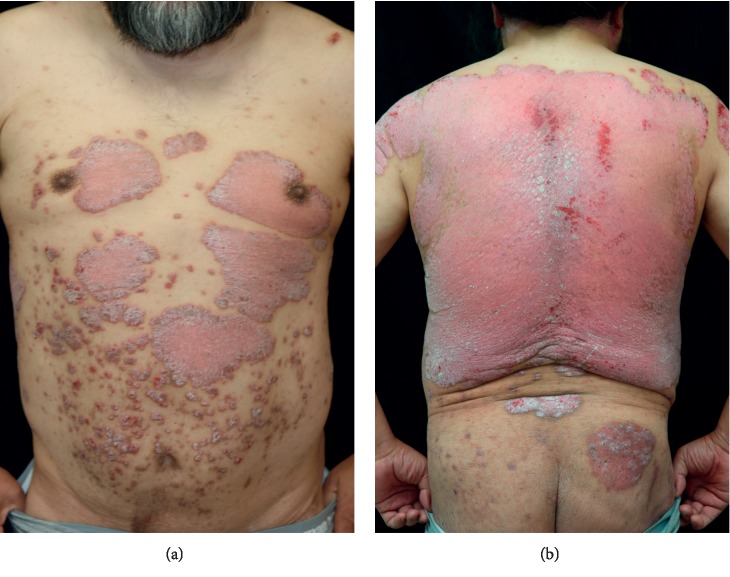
(a, b) Scaling erythematous plaques on the trunk before brodalumab treatment.

**Figure 2:**
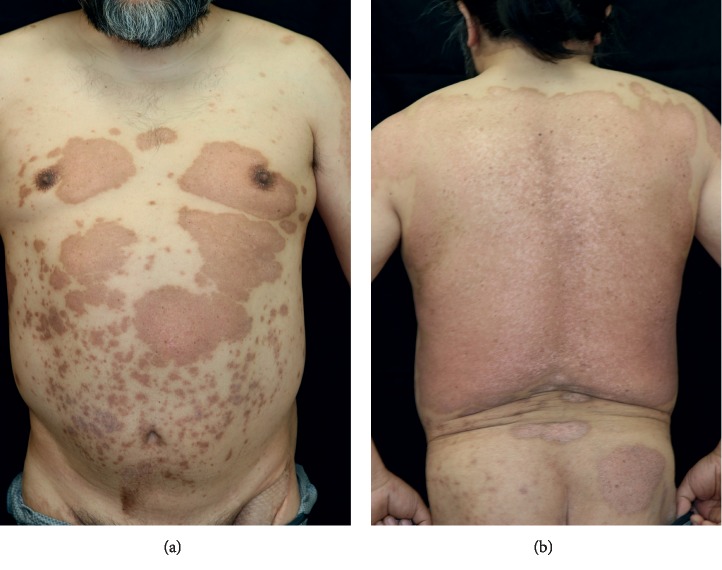
(a, b) Almost complete clearance of psoriatic lesions at 12 weeks after the start of brodalumab treatment.

**Figure 3:**
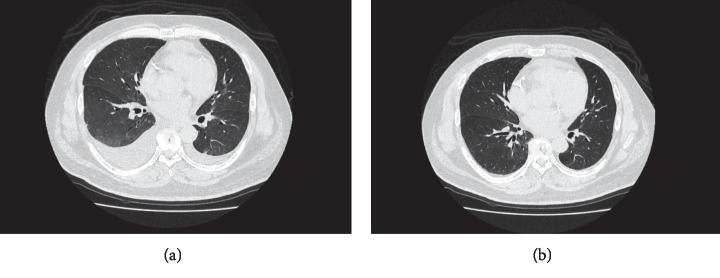
(a) Chest CT four months after brodalumab initiation, which showed bilateral ground-glass opacities and pleural effusions. (b) Seven months after brodalumab initiation, bilateral ground-glass opacities and pleural effusions improved.

A 60-year-old man was diagnosed with psoriasis 20 years ago for a widespread rash on the trunk. He was diagnosed with diabetes in his twenties and hypertension in his thirties but was untreated. He developed a necrotizing soft tissue infection of the thigh 12 years ago, and diabetes treatment was started. He was treated with cyclosporine 10 years ago at another hospital; this was discontinued due to renal dysfunction. Renal function gradually declined, and hemodialysis was introduced 2 months ago. During his visit to our department, psoriatic plaques were seen on his scalp, face, trunk, and limbs (Figures 1(a) and 1(b)). He had a Psoriasis Area and Severity Index (PASI) score of 39.6. In clinical examination, blood urea nitrogen was 48 mg/dL, serum creatinine was 11.57 mg/dL, and C-reactive protein was normal. Serum 1, 3-beta-D-glucan was negative. The T-SPOT.TB test was negative. He had no history of interstitial pneumonia and no specific findings on chest computed tomography (CT). He had received hemodialysis for diabetic nephropathy. He previously suffered from necrotizing soft tissue infection and had a high risk of developing infection. Although his skin lesion was severe, his adherence to the treatment was poor. Rapid onset of efficacy was considered to be important for treatment adherence. Treatment with brodalumab was initiated in terms of the efficacy and safety. After 4 weeks, the generalized rash was mainly pigmented; 100% improvement in the PASI score was achieved at 12 weeks (Figures 2(a) and 2(b)). He began to have a chronic cough four months after the start of the biologic treatment. He ceased his dermatology visit and had not been given brodalumab for a month. Coughing continued, and CT showed diffuse ground-glass opacities and pleural effusions in both lungs (Figure 3(a)). Transbronchial lung biopsy showed no findings suggestive of interstitial pneumonia. Grocott staining was negative in bronchoalveolar lavage fluid (BALF), as were acid-fast bacilli and fungal cultures. *Mycobacterium tuberculosis*, *Mycobacterium avium*, and *Mycobacterium intracellulare* were not identified by polymerase chain reaction in BALF. The T-SPOT.TB test was also negative. It was considered to be a symptom of overflow due to excessive fluid volume because of insufficient dietary management. Brodalumab was continued, and respiratory symptoms improved with proper weight setting and adequate dietary control. Chest CT showed improved pleural effusion and opacity (Figure 3(b)). No recurrence of rash has been seen twelve months after the initiation of brodalumab. There were no serious adverse events.

## 3. Discussion

Chronic kidney disease (CKD) is significantly associated with uncontrolled diabetes and hypertension. Some skin conditions are associated with CKD and end-stage renal failure (ESRD). Meanwhile, severe psoriasis is a risk factor for CKD and ESRD [6, 7]. Two cohort studies in Taiwan showed psoriasis was associated with nearly a 2- and 3-fold increased risk of CKD and ESRD, respectively. Serious infections increase among patients with psoriasis, and psoriasis is an independent risk factor for serious infections, according to studies from the Netherlands and the United States [8, 9]. Furthermore, the severity of psoriasis is suggested to be a predictor for the risk of severe infection [10], as with diabetes. With regard to the risk of infection among patients treated with biologics, respiratory and skin infections can occur under tumor necrosis factor *α* antagonist administration [11], and infliximab increases the risk of serious infections in psoriasis patients compared to nonbiological systemic therapies [12]. During anti-IL-17 therapy, the incidence of *Candida* infection increases only marginally, but no basis for an increased incidence of tuberculosis is found. A meta-analysis showed that there was no significant difference in the incidence of serious adverse events among patients treated with Th17 inhibitors versus placebo [13]. Respiratory diseases to be differentiated when using biologics are bacterial pneumonia, mycobacterial infection, pneumocystis pneumonia, and drug-induced interstitial pneumonia. Since hemodialysis patients are immunocompromised, regular monitoring of infections such as tuberculosis is necessary in those treated with IL-17 inhibitors, as with other biologics. Brodalumab is a highly efficacious therapy for psoriasis and may have the fastest onset of action of any biologic therapy currently available [14]. To our knowledge, this is the first reported case of psoriasis patients undergoing dialysis treated with brodalumab. It is suggested that brodalumab is useful for the treatment of severe psoriasis in patients undergoing hemodialysis, but more cases need to be reported for a better understanding.

## References

[1] Kusakari Y., Yamasaki K., Takahashi T. (2015). Successful adalimumab treatment of a psoriasis vulgaris patient with hemodialysis for renal failure: a case report and a review of the previous reports on biologic treatments for psoriasis patients with hemodialysis for renal failure. *The Journal of Dermatology*.

[2] Umezawa Y., Hayashi M., Kikuchi S. (2015). Ustekinumab treatment in patients with psoriasis undergoing hemodialysis. *The Journal of Dermatology*.

[3] Nimmannitya K., Tateishi C., Mizukami Y. (2016). Successful treatment with ustekinumab of psoriasis vulgaris in a patient undergoing hemodialysis. *The Journal of Dermatology*.

[4] Koike Y., Fujiki Y., Higuchi M., Fukuchi R., Kuwatsuka S., Murota H. (2019). An interleukin-17 inhibitor successfully treated a complicated psoriasis and psoriatic arthritis patient with hepatitis B virus infection and end-stage kidney disease on hemodialysis. *JAAD Case Reports*.

[5] Ikuma D., Oguro M., Hoshino J. (2019). Efficacy of secukinumab for plaque psoriasis in a patient on hemodialysis. *CEN Case Reports*.

[6] Chi C.-C., Wang J., Chen Y.-F., Wang S.-H., Chen F.-L., Tung T.-H. (2015). Risk of incident chronic kidney disease and end-stage renal disease in patients with psoriasis: a nationwide population-based cohort study. *Journal of Dermatological Science*.

[7] Yu S., Tu H.-P., Yu C.-L., Lee C.-H., Hong C.-H. (2017). Is psoriasis an independent risk factor of renal disease? A nationwide retrospective cohort study from 1996 to 2010. *Dermatologica Sinica*.

[8] Wakkee M., de Vries E., van den Haak P., Nijsten T. (2011). Increased risk of infectious disease requiring hospitalization among patients with psoriasis: a population-based cohort. *Journal of the American Academy of Dermatology*.

[9] Hsu D. Y., Gordon K., Silverberg J. I. (2016). Serious infections in hospitalized patients with psoriasis in the United States. *Journal of the American Academy of Dermatology*.

[10] Takeshita J., Shin D. B., Ogdie A., Gelfand J. M. (2018). Risk of serious infection, opportunistic infection, and herpes zoster among patients with psoriasis in the United Kingdom. *Journal of Investigative Dermatology*.

[11] Dixon W. G., Watson K., Lunt M. (2006). Rates of serious infection, including site-specific and bacterial intracellular infection, in rheumatoid arthritis patients receiving anti-tumor necrosis factor therapy: results from the British Society for Rheumatology Biologics Register. *Arthritis & Rheumatism*.

[12] Yiu Z. Z. N., Ashcroft D. M., Evans I. (2019). Infliximab is associated with an increased risk of serious infection in patients with psoriasis in the U.K. and Republic of Ireland: results from the British Association of Dermatologists Biologic Interventions Register (BADBIR). *British Journal of Dermatology*.

[13] Naik G. S., Ming W. K., Magodoro I. M. (2017). Th17 inhibitors in active psoriatic arthritis: a systematic review and meta-analysis of randomized controlled clinical trials. *Dermatology*.

[14] Papp K. A., Lebwohl M. G. (2017). Onset of action of biologics in patients with moderate-to-severe psoriasis. *Journal of Drugs in Dermatology*.

